# “Tree‐in‐bud” opacities with false‐positive Gaffky score and diffuse aspiration bronchiolitis

**DOI:** 10.1002/jgf2.374

**Published:** 2020-09-16

**Authors:** Mizuki Mogi, Hiroshi Hori, Takahiko Fukuchi, Hitoshi Sugawara

**Affiliations:** ^1^ Division of General Medicine Department of Comprehensive Medicine 1 Saitama Medical Center Jichi Medical University Saitama Japan

**Keywords:** diffuse aspiration bronchiolitis, Gaffky score, tree‐in‐bud opacities

## Abstract

We present a case of diffuse aspiration bronchiolitis (DAB) with a false‐positive Gaffky score. “Tree‐in‐bud” opacities detected after aspiration should be considered DAB rather than mycobacterial infection.

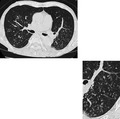

An 82‐year‐old man was transferred to our emergency department with symptoms of chills and 40.5°C fever. He vomited repeatedly and aspirated. His respiratory condition deteriorated, and he required oxygen therapy (10 L/min) to maintain SpO_2_ at 93%. Physical examination revealed bilateral coarse crackles at the base of the lungs. His serum C‐reactive protein (CRP) level was 4.74 mg/dL. Chest high‐resolution computed tomography (HRCT) revealed infiltrative shadows along the airway (Figure 1A), consistent with aspiration pneumonia. Blood and urinary cultures tested positive for *Aerococcus urinae*, suggesting a primary urinary tract infection (UTI) led to bacterial sepsis, followed by vomiting and aspiration pneumonia. He was treated with intravenous cefazolin 2 g per 6 hours for UTI with bacteremia. His respiratory condition initially improved, but he experienced clinical relapse, with a 38.0°C fever and CRP of 11.59 mg/dL, and resumed oxygen therapy. His fever persisted until day 9, when HRCT revealed centrilobular micronodules with “tree‐in‐bud” opacities throughout both lung fields (Figure 1B). He was isolated in a negative pressure room with suspected pulmonary tuberculosis. His sputum tested positive (Gaffky score 1) on one of three separate tests for *Mycobacterium* acid‐fast staining, but negative for *Mycobacterium tuberculosis, M avium,* and *M intracellulare* by transcription–reverse transcription concerted reaction (TRC). He was released from the negative pressure room after two negative acid‐fast tests, and another specimen was negative on TRC for *M tuberculosis*. An interferon‐γ release assay was also confirmed negative.

**Figure 1:**
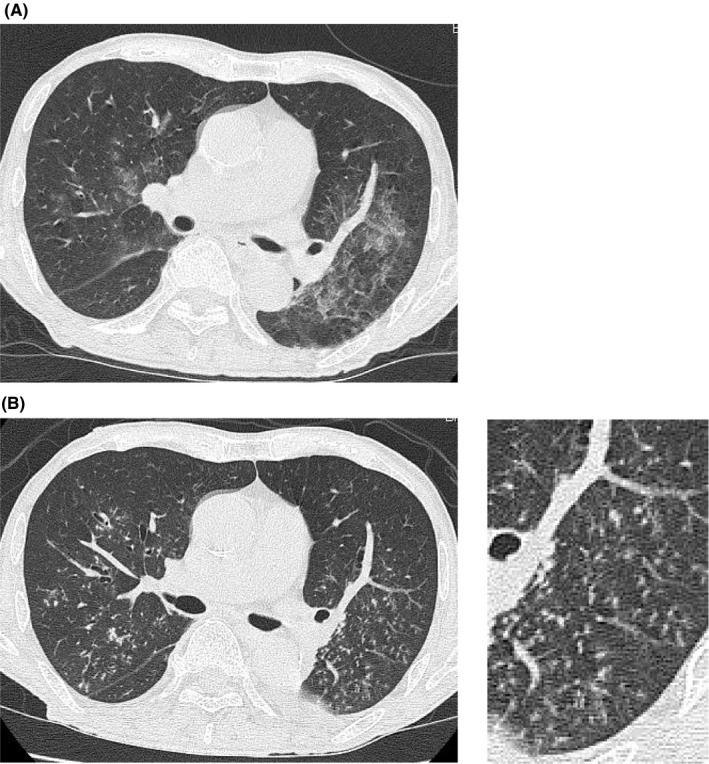
Computed tomographic images of the lungs. A, Computed tomography at admission shows notable infiltrative shadows along the airway in both lung fields. B, Computed tomography in hospital on day 9 shows left pleural effusion and centrilobular micronodules, as well as “tree‐in‐bud” branching opacities throughout both lung fields

His aspiration and HRCT indicated bronchiolitis, suggesting diffuse aspiration bronchiolitis (DAB). Treatment with sulbactam/ampicillin 3 g per 6 hours resulted in reduced fever and improved respiratory condition. One month later, his chest‐CT findings had disappeared (Figure 2) and a *Mycobacterium* sputum culture, incubated in both Ogawa and liquid mediums, tested negative.

**Figure 2:**
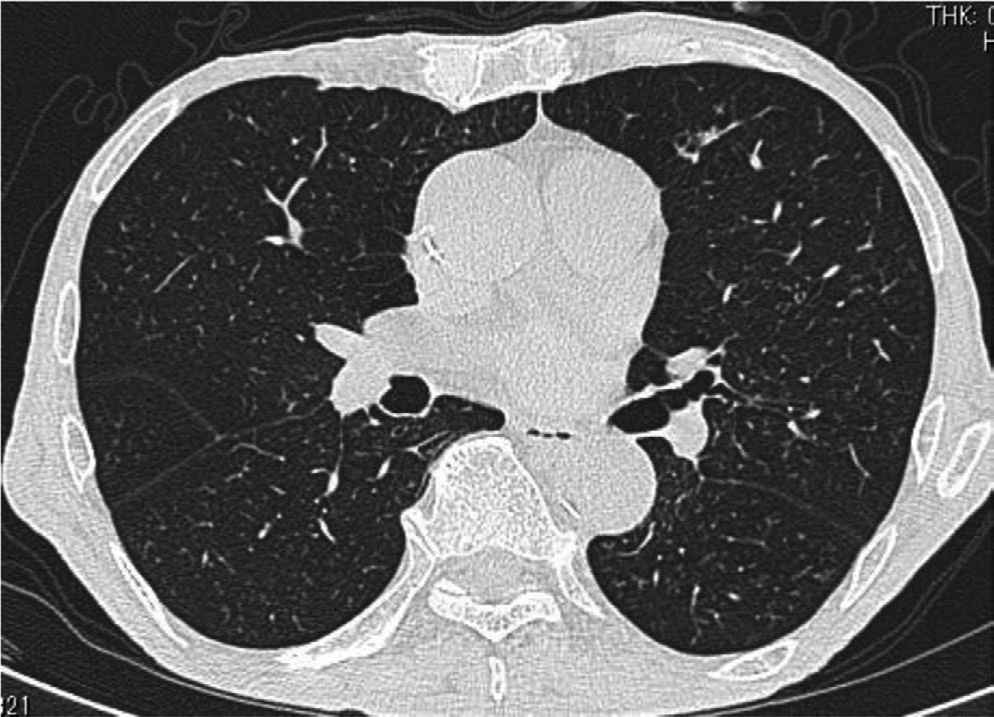
Computed tomography 1 month after hospital discharge shows that many centrilobular nodules with “tree‐in‐bud” branching opacities have disappeared

DAB is defined as chronic inflammation of bronchioles associated with recurrent aspiration of foreign materials.^1^ DAB has been identified in 16% of elderly patients with swallowing difficulties.^2^ Patients often have unremarkable symptoms and no abnormalities in objective swallowing function tests. HRCT typically reveals centrilobular micronodules (internal diameter ≤2 mm) with the classic “tree‐in‐bud” pattern throughout the lung fields. Pathological findings include multinucleated giant cells and granulomatous inflammation associated with foreign bodies.^3^

Differential diagnosis of the “tree‐in‐bud” pattern includes community‐acquired as acute disease, and mycobacterial pneumonia, diffuse panbronchiolitis, and DAB as subacute or chronic disease.

We tentatively suspected mycobacterial pneumonia based on “tree‐in‐bud” opacities on HRCT and *Mycobacterium* acid‐fast staining (Gaffky score 1). The low Gaffky score may have been secondary to the detection of mycobacteria other than *M* *tuberculosis* and findings related to food debris, digestive juices, and saliva.^4^ His clinical course indicated a diagnosis of suspected DAB from centrilobular micronodules and “tree‐in‐bud” branching opacities detected throughout both lung fields after aspiration. Patients with aspiration pneumonia are sometimes complicated with *Mycobacterium* infections, especially elderly patients. When physicians discover a “tree‐in‐bud” pattern, the patient should be placed in a negative pressure room, immediately followed by acid‐fast bacilli smears and cultures in three separate tests.^5^ DAB should also be considered as a differential diagnosis.

## CONFLICT OF INTERESTS

The authors have stated explicitly that there are no conflicts of interest in connection with this article.

## INFORMED CONSENT

We have obtained written patient consent for publication of this case report.
